# Medical dissolution of presumptive upper urinary tract struvite uroliths in 6 dogs (2012‐2018)

**DOI:** 10.1111/jvim.17204

**Published:** 2024-10-05

**Authors:** Sindumani A. Manoharan, Allyson C. Berent, Chick W. Weisse, Kira Purdon, Demetrius Bagley

**Affiliations:** ^1^ Department of Diagnostic Imaging and Interventional Radiology The Animal Medical Center New York New York USA; ^2^ Department of Urology Jefferson Medical College, Thomas Jefferson University Philadelphia Pennsylvania USA

**Keywords:** ammonium, antibiotic, canine, diet, infection, magnesium, phosphate

## Abstract

**Background:**

Minimally invasive approaches are the standard for treatment of upper urinary tract uroliths in humans.

**Objective:**

To describe the medical dissolution of upper urinary tract uroliths in a series of dogs and report clinical outcomes.

**Animals:**

6 female dogs (9 kidneys).

**Methods:**

Retrospective case series. A review of medical records in dogs that underwent medical dissolution of upper urinary tract uroliths utilizing diet, administration of antibiotics, and double‐pigtail ureteral stent(s) placement, when indicated, was performed. Medical management was generally continued for 4 weeks beyond urolith dissolution. Information on biochemical, microbiological, imaging, and clinical outcomes before and after dissolution were recorded.

**Results:**

Six dogs (9 kidneys) were included with bilateral (3) or unilateral (3) nephrolithiasis, ureterolithiasis, or a combination. A ureteral stent(s) was placed endoscopically in 5/6 dogs (6/9 kidneys) for obstructive ureterolithiasis (n = 5) or a nonobstructive massive nephrolith (n = 1). All dogs had a positive urine culture of *Staphylococcus pseudintermedius* with a median urine pH of 7.25 (range, 6.5‐8) and 4/5 had pyonephrosis. All dogs had initial evidence of urolith dissolution at a median of 1.1 months (range, 0.42‐5.9), with complete dissolution of ureteroliths at a median of 3.9 months (range, 1.5‐7.6), nephroliths at 5.3 months (range, 1.5‐7.6), and lower urinary tract uroliths at 0.87 months (range, 0.42‐5.9). Stents were removed in 3/6 once dissolution was documented. The median follow‐up time was 519 days (range, 177‐2492 days).

**Conclusion and Clinical Importance:**

Medical dissolution and decompression of upper urinary tract struvite uroliths should be considered a minimally invasive treatment for dogs before more invasive options.

**Abbreviations**
BUN: blood urea nitrogen
ENL: endoscopic nephrolithotomy
ESWL: extracorporeal shockwave lithotripsy
HPF: high‐power field
UPJ: ureteropelvic junction
UTI: urinary tract infection

## 1 INTRODUCTION

Struvite uroliths, a combination of magnesium, ammonium, and phosphate, are a common cause of urinary calculi in dogs.^1^ Despite their potential to be dissolved with diet and antibiotics in dogs, many cases are still surgically managed.^2^, ^3^, ^4^ Struvite uroliths, often linked to urinary tract infections (UTIs) and more prevalent in female dogs, are colloquially termed “infection‐associated uroliths.”^5^, ^6^ Although certain breeds are predisposed, any dog with infected urine containing a urease‐producing organism, such as *Staphylococcus pseudintermedius*, *Proteus* spp, *Pseudomonas* spp, and *Klebsiella* spp, is at risk.^7^

In a recent series, obstructive pyonephrosis in 13 dogs was treated with ureteral stenting and renal pelvic lavage, effectively dissolving suspected struvite nephrolithiasis after stenting with appropriate diet and antibiotic treatment.^8^ Treatment of nephroureterolithiasis in dogs is limited in veterinary literature.^9^, ^10^, ^11^ Traditional surgical options include nephrotomy, ureterotomy, ureteronephrectomy, neoureterocystostomy, or pyelotomy^12^, ^13^, ^14^, ^15^, ^16^ while more minimally invasive options such as extracorporeal shockwave lithotripsy (ESWL),^2^, ^17^, ^18^ endoscopic nephrolithotomy (ENL)^2^, ^19^, ^20^ and percutaneous fluoroscopic‐assisted and retrograde ureteral stenting are also available.^8^, ^21^, ^22^ In humans, anterograde stenting via percutaneous access is used in emergency settings, but this technique carries less risk compared to veterinary dogs because of a larger and more stable retroperitoneal kidney, and ability to access the renal pelvis without transversing the peritoneum.^23^, ^24^, ^25^, ^26^, ^27^, ^28^, ^29^ However, in dogs, retrograde stenting is considered safer because of lower risk for septic uroabdomen. Open surgical approaches in small animals carry substantial complications such as bleeding, nephrolith or edema‐associated persistent ureteral obstructions, urinary leakage, urosepsis, and progressive renal functional loss.^2^, ^14^ In humans, minimally invasive techniques are considered the standard of care for treatment of upper urinary tract uroliths, as they are associated with improved postoperative recovery, minimal renal functional loss, fewer operative and postoperative complications, and shortened hospital stays.^2^, ^23^, ^24^, ^25^, ^26^, ^27^, ^28^, ^29^

While efficacy of medical dissolution of upper urinary tract uroliths has been questioned, recent studies report success with ureteral stenting.^2^, ^8^, ^21^ Ureteral stenting in dogs is a safe and highly effective minimally invasive treatment option for the treatment of ureteral obstructions and is associated with the lowest perioperative morbidity and fatality rates reported to date in the limited veterinary literature.^8^, ^15^, ^21^ Regardless of the technique chosen, avoiding renal parenchymal access preserves the most renal function and reduces risk.^6^, ^8^, ^19^, ^30^, ^31^, ^32^, ^33^, ^34^ As struvite nephroureterolithiasis often involves pyonephrosis and urosepsis, and over 40% of dogs have associated moderate to severe thrombocytopenia, likely related to their sepsis,^8^, ^15^, ^21^ open abdominal surgery is less appealing.^35^ Diet and targeted antibiotics can effectively treat struvite urolithiasis as diet can be readily controlled in dogs.^2^, ^36^, ^37^, ^38^, ^39^

The objective of our study was to describe a case series where medical dissolution was utilized for the treatment of upper urinary tract nephroureterolithiasis caused by suspected struvite uroliths. The hypothesis was that upper urinary tract struvite uroliths are highly responsive to medical dissolution, avoiding invasive and complicated surgeries, especially in the presence of urosepsis or infected urine, and can be associated with a successful outcome.

## 2 MATERIALS AND METHODS

### 2.1 Case selection

Medical records from the Animal Medical Center (AMC), New York, were electronically searched to identify dogs evaluated for nephrolithiasis, ureterolithiasis, or a combination associated with suspected struvite uroliths. Struvite urolith type was determined either through qualitative urolith analysis, or predicted based on a combination of a fasting urine pH of >7.0, bacteriuria, a urine culture consistent with a urease‐producing organism (eg, *Staphylococcus* spp, *Proteus* spp, or *Klebsiella* spp), and radiopaque uroliths, commonly staghorn, seen on radiographic images. Cases were included if they had ultrasonographic and radiographic imaging before dissolution, biochemical (complete blood count and blood chemistry) and urine microbiological (urinalysis and urine culture) data before treatment, and serial follow‐up imaging, biochemical, and microbiological data. Radiographic imaging was reviewed monthly when available during follow‐up. Cases were excluded if the dissolution diet and antibiotic protocol were not followed, or records were not available for review as described.

### 2.2 Medical records review

Data were retrospectively collected from the medical records. This included data on signalment, clinical signs and physical examination at initial evaluation, historical findings (including evidence of prior UTIs and previous urolithiasis), concurrent illness, results of urinary tract ultrasonography, and abdominal radiography when available, results of current clinicopathologic tests (urinalysis, aerobic urine culture and susceptibility, complete blood count, serum biochemical analyses, urolith analysis), type and size of ureteral stent used when indicated, procedure report details, duration of stenting procedure, intraoperative, perioperative, and postoperative complications, duration of hospitalization, survival, and long‐term serial follow‐up information. Long‐term follow‐up included data on serial serum biochemical analysis, urinalysis and aerobic bacterial culture and sensitivity results, imaging, concurrent medications utilized, diet, and final clinical status at the time of last follow‐up.

### 2.3 Dissolution protocol

The standard dissolution protocol included an appropriate antibiotic choice based on microbiologic sensitivity testing at therapeutic dosages, a dissolution/neutralizing diet, and increasing water intake to a goal urine specific gravity of <1.020. Dietary regimen was either a prescription dissolution diet (Urinary SO, Royal Canin Veterinary Diets, St. Charles, Missouri; C/D Multicare, Hill's Prescription Diets, Topeka, Kansas) or a homemade diet prepared by a nutritionist for struvite urolith dissolution. Traditionally, dogs were recommended a moist diet with additional water added to encourage polydipsia and promote more frequent elimination.

## 3 PROCEDURES

Pre‐operative preparation of the dog is described Data S1. Dogs with a concurrent ureteral obstruction had a double‐pigtail ureteral stent placed using endoscopic and fluoroscopic guidance as previously described.^8^, ^10^, ^21^ The procedure time and any complications with stent placement were reported. If pyonephrosis was present, the renal pelvis was lavaged during a retrograde ureteropyelogram, before stent placement and the urine from the renal pelvis or ureter was cultured, as described elsewhere.^8^

### 3.1 After procedural management

Each dog continued on full dose antibiotics and dissolution diet for 1 month beyond the visible resolution of urinary urolith fragments based on imaging. Urine culture obtained via cystocentesis was recommended 7 to 10 days after the 1st administration of antibiotics and then monthly. Radiographs were evaluated monthly to ensure the uroliths were dissolving, and ultrasonography was repeated at 1 month and every 3 months to check stent placement and renal pelvis decompression. When the urolith was completely dissolved, the ureteral stent was typically removed endoscopically. One month after complete dissolution, the dog was transitioned to a maintenance diet and antibiotics were discontinued. Follow‐up urine cultures were recommended 2‐4 weeks later and then every 3 months to monitor for bacteriuria recurrence.

## 4 RESULTS

### 4.1 Clinical data

Six dogs, and 12 kidneys, were assessed during medical record review. All dogs were spayed females including the following breeds: miniature schnauzer (n = 2), and 1 each of Yorkshire terrier, shih tzu, Lhasa apso, schnauzerpoo, and a Pembroke Welsh corgi. The median age was 5 years (range, 4‐10) and the median weight was 7.41 kg (mean, 7.94; range, 3.42‐12.86). Concurrent medical problems included: transitional cell carcinoma, degenerative mitral valve disease, chronic lower airway disease, an ectopic ureter, diabetes mellitus, pancreatitis, collapsing trachea, caudal occipital malformation syndrome, and a previous infection with *Anaplasma phagocytophilum*. Presenting clinical signs included decrease appetite (5/6), abdominal pain (5/6), hematuria (4/6), vomiting (5/6), dysuria (4/6), lethargy (4/6), and fever (2/6). The duration of systemic signs was reported to be a median of 2 days (range, 1‐4). The duration of lower urinary tract signs was a median of 1 day (range, 1‐5).

Two of 6 dogs had a prior history of cystolithiasis and treatment included a cystotomy and dissolution diet with antibiotics in 1 dog 259 days before presentation. The urolith type analyzed via crystallographic analysis from the cystotomy of this dog was a mix of magnesium ammonium phosphate with a calcium phosphate carbonate center. The urolith analysis from 1 of these 2 dogs was unavailable. The median duration of known nephroureterolithiasis before presentation was 2.5 days (range, 1‐76).

At the time of presentation, a urinalysis was performed in all 6 dogs with a median urine pH of 7.25 (range, 6.5‐8, reference range, 6.5‐7.0). Two dogs had struvite crystalluria, 2 pyuria (median 0‐2/high‐power field (HPF), range, 0‐>100, reference range, 0‐5), 6 hematuria (median >100 per HPF, range, 2‐5 to 100, reference range, 0‐5), and 2 had bacteriuria with cocci observed.

The urine bacteriologic culture and sensitivity results were obtained by cystocentesis in all 6 dogs. The results were positive in 5 dogs tested off antibiotics for *S pseudintermedius* and negative in 1 dog that was on antibiotics before presentation. One dog was positive at the time of presentation to the AMC and 3 others were positive 48, 54, and 259 days before at the primary veterinarian. One dog was hospitalized with antibiotic treatment at another emergency facility 7 days before referral without any culture during this episode, but was historically positive. Two dogs that were positive had a susceptibility resistance pattern against penicillins. Five dogs had a prior history of a UTI with *S pseudintermedius* by sterilely obtained urine culture a median of 54 days before presentation (range, 2‐301 days) and all were treated with amoxicillin/clavulanic acid. One dog with a negative urine culture obtained by cystocentesis at the time of presentation had evidence of pyonephrosis on ultrasonography. However, submission of renal pelvis urine for culture was declined for financial reasons. No dog had a positive renal pelvis culture with a negative urine culture via cystocentesis.

### 4.2 Diagnostic imaging

After reviewing both abdominal radiographs (5 dogs) and ultrasonography images (6 dogs), 9 of 12 kidneys were affected. Eight of 12 kidneys had a nephrolith and 5 of 12 had a ureterolith, all 5 of which were obstructive based on ultrasonographic findings. Ureteral obstruction was suspected when renal pelvic dilatation was documented with an associated hydroureter to an obstructive lesion. Each obstruction was ultimately confirmed with a retrograde ureteropyelogram during ureteral stent placement. Four of 6 dogs had a cystolith(s), 1 had a urethrolith, and 1 had mineralized bladder sediment. Four of 6 dogs had evidence of pyonephrosis based on presence of echogenic material in renal pelvis (range, 0.39‐7.2 cm), and when a ureteral stent was placed retrograde, the renal pelvic lavage and pyelocentesis documented purulent material from the renal pelvis.

In the 5 dogs with radiographs, 3 had nephroliths, 4 had ureteroliths, and 2 had cystoliths identified. No dog had urethroliths identified with radiographs. In the 6 dogs with ultrasonography imaging, 5 had nephroliths, 5 had ureteroliths, 3 had cystoliths, 1 had mineralized sediment, and 1 had urethroliths identified. The median ureteral urolith diameter as measured on the ventrodorsal projection in the longitudinal by transverse plane was 0.96 cm (range, 0.56‐2.5 cm). The size of the nephroliths were not able to be reliably measured with this modality.

Radiographs available for 5 dogs (10 kidneys) identified ureteroliths in 4/10 kidneys, nephroliths in 4/10, 2 dogs with cystoliths, and 1 with urethroliths. Ultrasonography available for 6 dogs (12 kidneys) identified ureteroliths in 5/12 kidneys, nephroliths in 8/12 kidneys, 3 dogs with cystoliths, 1 dog with urethroliths, and 1 dog with mineralized sediment.

On ultrasonography, the median ureteral and kidney urolith diameters were 0.85 cm (range, 0.6‐1.6 cm) and 1.0 cm (range, 0.2‐7.2 cm), respectively. Other imaging findings included evidence of a retroperitoneal abscess caudal to the obstructive right kidney and pancreatitis in 1 dog, and a suspected chronic hematoma or organized solidified purulent mass seen in the renal pelvis of 1 dog, which could not be identified on radiographs.

**FIGURE 1:**
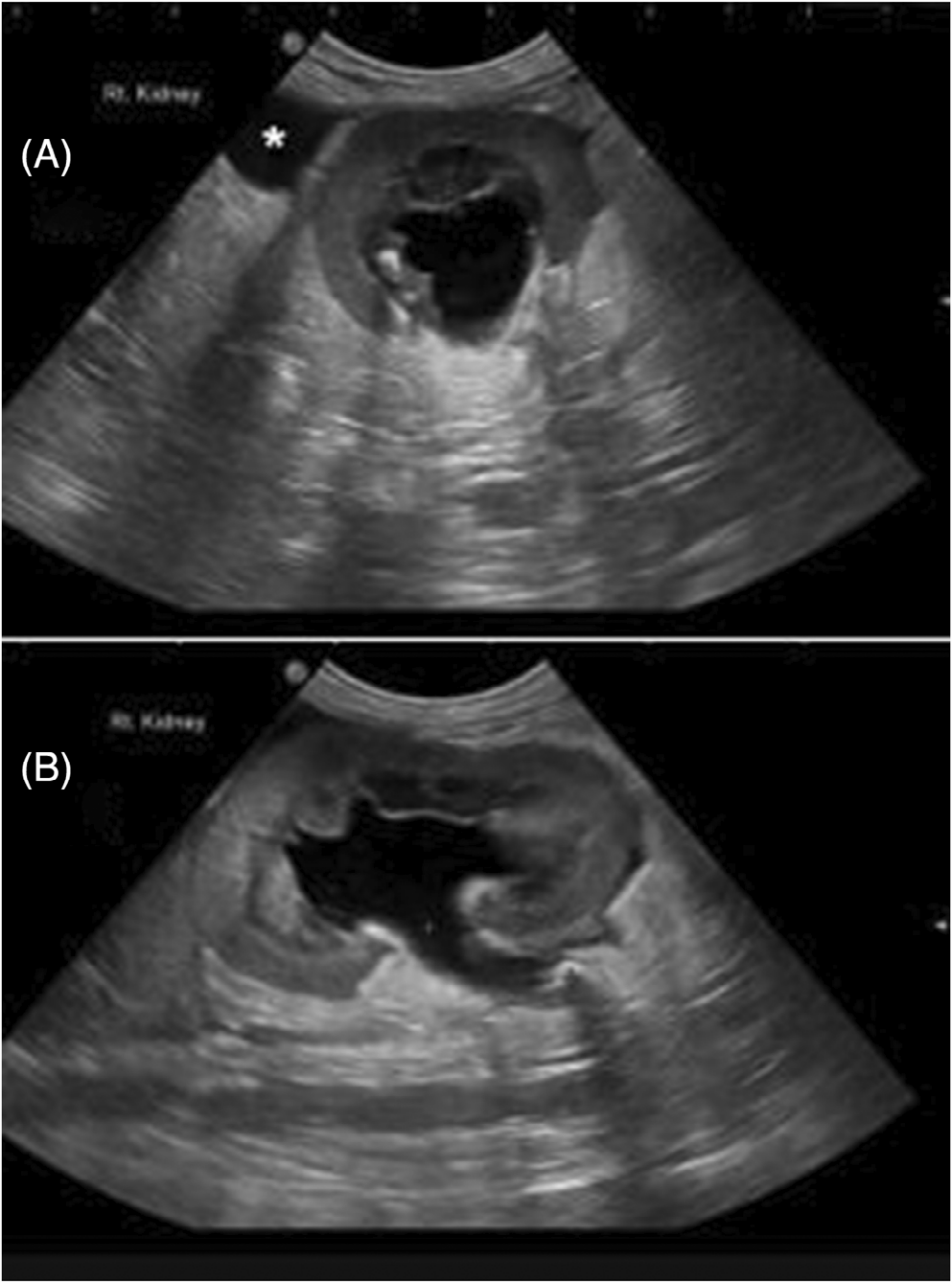
Ultrasonographic image of dilated renal pelvis with associated pyonephrosis and perinephric abscess* (A) Short axis transverse. (B) Long axis sagittal.

Overall, 5/6 dogs had evidence of a unilateral ureteral obstruction(s) secondary to a ureterolith. Concurrent hydronephrosis and hydroureter (4 right, 1 left) was present in all dogs associated with the ureterolithiasis, and 3 of 5 obstructed kidneys had ultrasonographic evidence of echogenic material in the renal pelvis consistent with pyonephrosis. The median dilatation of the renal pelvis in the transverse plane was 2.1 cm (range, 0.39‐7.2 cm) (Figure 1). The median size of the ureteral dilatation was 0.7 cm (range, 0.22‐1.6 cm**).**

Radiographs identified 1 dog that had urinary bladder uroliths outside of the bladder lumen from a previous cystotomy that were not identified on ultrasonography. Ultrasonography identified 2 dogs with nephroliths, 1 dog with ureterolith and cystolith, and 1 dog with mineralized bladder sediment that were missed on radiographs.

Pelvic dilatation was observed in 3 kidneys without a concurrent ureteral obstruction. Two dogs had renal pelvis dilatation caused by nephroliths, with no associated hydroureter or ureteral uroliths. One dog with a unilateral nephrolith and obstructive ureterolith in the ipsilateral kidney also had renal pelvic dilatation (0.39 cm) in the contralateral kidney, without nephroliths or ureteroliths to explain the dilatation. Ureteral dilatation (0.22 cm) was seen in 1 dog with no ureteral calculi on ultrasonography or radiographs.

### 4.3 Procedures

Of the 6 dogs in the study, 6 double‐pigtail ureteral stents were placed in 5 dogs because of obstructive ureterolithiasis in 5 and a massive nonobstructive nephrolith (7.2 cm) in a contralateral kidney in 1. Five ureters in 5 dogs (5/12 kidneys) had evidence of a ureteral obstruction and a stent was placed endoscopically in all obstructed kidneys. Four of 5 obstructed kidneys showed associated pyonephrosis during sampling. One dog with pyonephrosis intraoperatively had no appreciable echogenic debris detected on the prior abdominal ultrasonography. The 1 dog that did not have a ureteral stent placed had nonobstructive nephroliths (0.9 cm) in both kidneys not requiring stent placement.

All stents were successfully placed endoscopically using endoscopic and fluoroscopic guidance (1.9‐mm rigid 30° cystoscope, Karl Storz Endoscopy, Culver City, California; 2.7‐mm rigid 30° cystoscope, Karl Storz Endoscopy, Culver City, California). The median ureteral stent size used was 4.7 Fr × 20‐30 cm variable length (range, 3.7‐4.7Fr, 16‐32 cm) (4.7Fr × 20‐cm double‐pigtail ureteral stent, Infiniti Medical LLC, Menlo Park, California; 4.7Fr × 22‐32‐cm variable‐length double‐pigtail ureteral stent, Bard Medical, Covington, Georgia; 5Fr × 22‐32‐cm variable‐length double‐pigtail ureteral stent, Cook Medical, Bloomington, Illinois; 7Fr × 16 cm double‐pigtail ureteral stent, Cook Medical, Bloomington, Illinois.). The median procedure time was 45 minutes (range, 35‐75 minutes). Two dogs had an esophagostomy tube placed for nutritional support during recovery, which was included in the surgical time. No dog required conversion to open surgery for ureteral stent placement.

### 4.4 Complications

**FIGURE 2:**
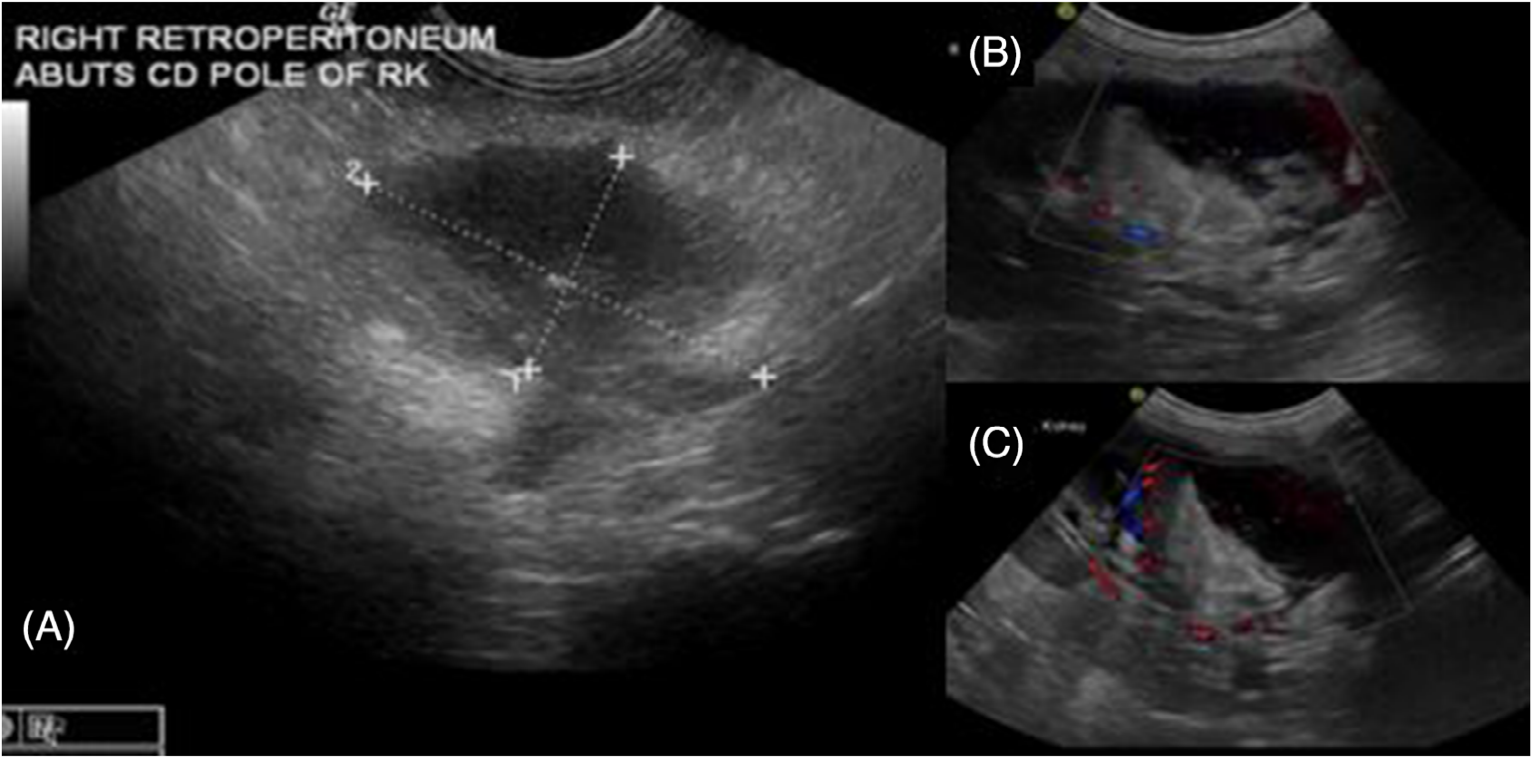
Right retroperitoneum and caudal pole of right kidney. (A) Initial retroperitoneal abscessation on presentation. (B) and (C) Progressive retroperitoneal abscessation and suspected focal area of renal calyceal rupture associated with ureteral obstruction.

In 2 dogs, the guide wire penetrated the ureteral wall at the obstruction site, causing contrast extravasation. However, this did not affect treatment, as the stent was successfully placed endoscopically after redirecting the wire. Another dog experienced weakness, apparent nausea, and lethargy after the procedure, without procedural complication. A subsequent ultrasonography revealed progressive retroperitoneal abscess formation, noted on initial presentation (Figure 1), around the caudal pole of the obstructed kidney (Figure 2). This was considered a focal area of renal calyceal rupture that had sealed at time of retrograde ureteropyelogram. Percutaneous drainage was performed under ultrasonographic guidance, leading to rapid improvement. The dog was discharged the next day with enrofloxacin administered orally, and then ultimately transitioned to subcutaneous meropenem.

### 4.5 Management

The median blood urea nitrogen (BUN) and creatinine concentration obtained before discharge was 14 mg/dL (range, 4‐43; reference range, 9‐31) and 1.1 mg/dL (range, 0.6‐2.4; reference range, 0.5‐1.5), respectively. Five dogs were hospitalized for medical management and ureteral decompression, with a median hospital stay of 2 days (range, 0‐6). One dog was managed on an outpatient basis for bilateral nonobstructive nephroliths.

Antibiotics were prescribed for all dogs, with 3 prescribed enrofloxacin (10.9‐15.2 mg/kg PO q24h), 2 prescribed a combination of enrofloxacin (10.6‐15.5 mg/kg PO q24) and amoxicillin/clavulanic acid (14.8‐16.9 mg/kg PO q12h), and 1 with amoxicillin/clavulanic acid (18.2 mg/kg PO q12), pending sensitivity results. One dog was administered meropenem (SC) after return of sensitivity results.

All dogs were discharged with a prescription diet (Urinary SO, Royal Canin Veterinary Diets, St. Charles, Missouri; C/D Multicare, Hill's Prescription Diets, Topeka, Kansas), and 1 dog was transitioned to homemade dissolution diet after consultation with veterinary nutritionist. Other medications administered at home included the following: omeprazole (0.5‐1 mg/kg PO q12h), maropitant (2 mg/kg PO q24h), capromorelin (3 mg/kg PO q24h), codeine (1‐2 mg/kg PO q8h), famotidine (0.5 mg/kg PO q12h), and ondansetron (0.2‐0.5 mg/kg PO q12h).

### 4.6 After operative clinical data

All dogs received antibiotic treatment, adjusted based on sensitivity results. One dog received amoxicillin/clavulanic acid exclusively, 3 were treated with enrofloxacin alone, and 2 received a combination of amoxicillin/clavulanic acid and enrofloxacin. One dog initially prescribed enrofloxacin was transitioned to meropenem (SC) because of evidence of pyonephrosis despite a negative bladder urine culture. This dog later was prescribed nitrofurantoin and enrofloxacin because of concurrent multidrug‐resistant *E coli* detected during dissolution period. Another dog initially on combination treatment was changed to cefpodoxime because of financial constraints. A 3rd dog transitioned from enrofloxacin to cefpodoxime because of persistent infection. During the dissolution period, 3 dogs developed new UTIs with different organisms, including *S schleferi*, *E coli*, or *Enterococcus*. Treatment was tailored to address both the new infections and the primary *S pseudintermedius* infection that caused the struvite uroliths, ensuring dissolution continued. Throughout the dissolution period, no dogs exhibited resistance to *S pseudintermedius*.

One of 2 dogs with documented azotemia at the time of presentation had normalization of BUN and creatinine after ureteral decompression, and 1 dog remained mildly azotemic at the 1‐month after stent examination (creatinine 2.5 mg/dL). The median creatinine concentration for all dogs on follow‐up at 1 month was 1.2 mg/dL (range, 1‐2.5), and at the time of last follow‐up at 14.5 months (range, 5‐65) was 1.1 mg/dL (range, 0.9‐1.2).

The median time for lower urinary tract signs to resolve was 1.5 days (range, 1‐4 days). Two dogs continued to have polyuria and polydipsia, 1 of which also had diabetes mellitus.

All dogs had initial documentation of upper urinary tract urolith dissolution at a median of 1.1 months (range, 0.42‐5.9). All dogs (n = 6) had complete dissolution of ureteroliths at a median of 3.9 months (range, 1.5‐7.6), nephroliths at 5.3 months (range, 1.5‐7.6), and cystoliths and urethroliths at 0.87 months (range, 0.42‐5.9). No dog had their cystoliths or urethroliths removed, as they all dissolved.

**FIGURE 3:**
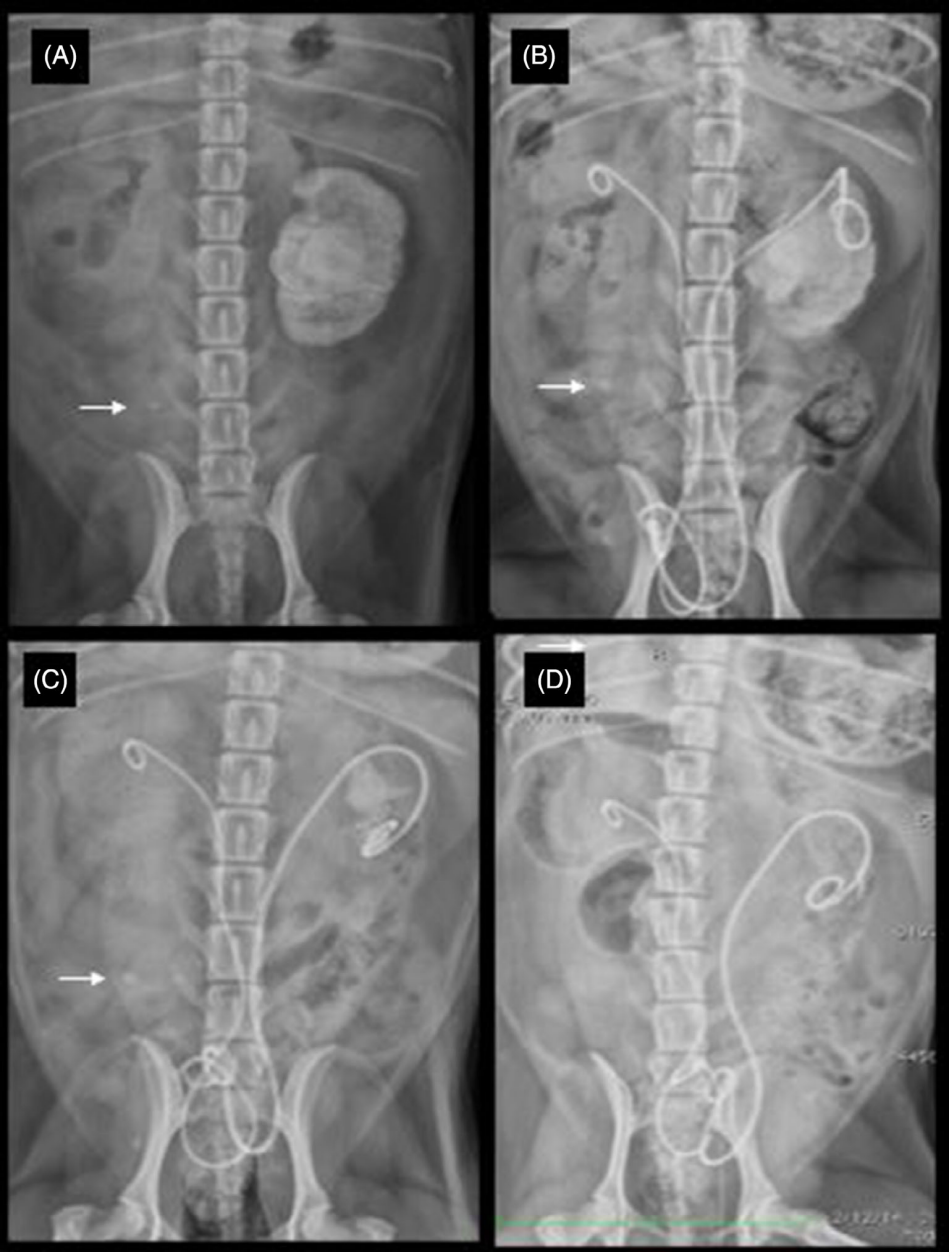
Radiographic images of a female dog in dorsal recumbency with bilateral double‐pigtail ureteral stent placement. (A) Right obstructive ureterolith (white arrow) and ipsilateral nephrolith and contralateral nonobstructive massive nephrolith. (B) Three months after dissolution and decompressive bilateral stent placement. (C) Five months after dissolution and decompressive stent placement. (D) Eight months after dissolution and decompressive stent placement.

Complete upper urinary tract urolith dissolution was achieved for all ureteroliths (n = 5) and 7/8 nephroliths. The 1 dog without complete dissolution of the nephrolith had development of cystic calculi at 13.2 months, and a newly appreciated radiolucent ureterolith in the contralateral ureter to its previously obstructive ureterolith 31.8 months after initiation of a dissolution diet. This dog had recurrent UTIs and had a ureteral stent exchange at 13 months after placement because of persistent renal pelvis fragments. At this time, this dog had small bladder uroliths analyzed and the fragments were consistent with calcium apatite. This same dog had complete dissolution of obstructive ureterolith, ipsilateral nephrolith, and over 95% of the contralateral nephrolith, which was 7.2 cm in length at the start of dissolution (Figure 3). This is the same dog that was presented with a retroperitoneal abscess from presumptive renal pelvic rupture. At follow‐up, there were only small fragments remaining after 9 months of dissolution diet and antibiotics and the fragments did not progressively dissolve, so were assumed to be calcium apatite like the bladder uroliths. One dog had recurrent right nonobstructive nephroliths 145 days after dissolution of the initial uroliths. This dog also had a recurrent UTI with *S pseudintermedius* and did not have a ureteral stent at the time of rediagnosis because of the lack of ureteral obstruction. Reinstitution of a dissolution strategy was employed.

Three of 6 ureteral stents placed were removed endoscopically without complications after dissolution of all upper urinary tract uroliths at a median time of 231 days after placement (range, 143‐399). One dog had a unilateral ectopic ureter ablation at the time of stent removal, which was diagnosed incidentally during initial stent placement. Because of concurrent UTI, the ectopic ureter was not corrected until a negative urine culture was documented and a rescoping was planned. The dog that had a ureteral stent placed because of the massive nonobstructive nephrolith had 2 stent exchanges to that kidney because of recurrent UTIs and suspected pyelonephritis. Intraoperative antibiotics based on prior urine culture and sensitivity, and serial urine monitoring was encouraged to minimize inoculation of new stent during exchange. The contralateral stent had been removed once all urolith fragments dissolved, but the nephrolith had remaining fragments. Despite recommendations for stent removal, the owner elected to keep the stent in place as there were no clinical signs and the owner had grave concern for the development of a future obstruction. Two dogs had their ureteral stents remain in place despite resolved urolithiasis because of poor follow‐up and compliance after dissolution was documented.

### 4.7 Outcome

The median time to last follow‐up was 519 days (range, 177‐2492 days) with 4 of 6 dogs still alive at the time of writing this manuscript. The cause of death in the 2 dogs was transitional cell carcinoma at 178 days after presentation in 1 with new ultrasonographic changes within urinary bladder and compatible urine cytospin, and unknown but unrelated to renal or ureteral disease in the other at 1424 days after presentation. One dog had evidence of urolith recurrence during the follow‐up period in the contralateral ureter. A total of 3 dogs developed additional clinical UTIs during the dissolution period, 1 with a combination of *S schleferi* and *Enterococcus* spp., and 2 with an *E coli* (both had a ureteral stent and 1 also had a concurrent contralateral ectopic ureter). After urolith dissolution and stent removal, 1 of those 3 dogs developed another UTI with *Enterococcus* spp. and *E coli*. One of these 3 dogs had 2 ureteral stent exchanges at 13 and 35 months, respectively, because of chronic, recurrent *E coli* infections and persistent nonstruvite nephroureterolithiasis that were not amendable to medical dissolution. Another dog with complete dissolution of right nephro‐ureterolith had UTI recurrence with a multidrug‐resistant *S pseudintermedius*, and associated urolith recurrence (small right renal and cystic calculi) 12.3 months after initial urolith dissolution and discontinuation of antibiotics.

## 5 DISCUSSION

In our study involving 6 dogs, the effectiveness of medical dissolution treatment for suspected struvite upper urinary tract uroliths was supported. All dogs were female spayed small breeds, with *Staphylococcus* infections, alkaline urine pH, radio‐opaque uroliths on radiographs, and associated ureteral obstruction with pyonephrosis. All dogs in our study had successful dissolution of their upper and lower uroliths. Treatment included combination of diet, antibiotics, and ureteral stents when obstruction was confirmed. Ureteral stents were placed minimally invasively in all obstructed ureters (n = 5) using endoscopic and fluoroscopic guidance. Of the kidneys with uroliths (7 nephroliths and 5 ureteroliths), 8/9 were completely dissolved with diet and antibiotics. One dog had incomplete dissolution of a nephrolith, with residual fragments consistent with calcium apatite, suggesting they were not struvite. This dog had a large nephrolith (>7 cm), with remaining fragments under 3‐5 mm (Figure 3).

In the present study, the most common presenting clinical signs were abdominal pain (5/6), decreased appetite (5/6), vomiting (5/6), lethargy (4/6), dysuria (4/6), hematuria (4/6), and increased rectal temperature (2/6). Historical microbial culture of 5 of 6 dogs indicated evidence of a UTI with *S pseudintermedius*, all of which were treated with amoxicillin/clavulanic acid. A microbial culture collected via cystocentesis was repeated in all dogs with upper urinary tract uroliths upon evaluation and before ureteral stent placement when indicated and 3 of 6 had an active infection of *S pseudintermedius*, despite 2 of 3 dogs being initiated on broad‐spectrum coverage of antibiotics before admission.

In the present study, all dogs underwent similar diagnostic imaging procedures to diagnose and localize uroliths, and the combination of abdominal radiography and ultrasonography was the most accurate representation of urolith location, size, opacity, and obstructive nature.

Ultrasonography found pelvic dilatation in 3 dogs without ureteral obstruction. In 1 dog with a nephrolith and ipsilateral ureterolith, contralateral renal pelvic dilatation was seen without uroliths. Given absence of obstructive lesion, the combination of pyelonephritis within this kidney with contralateral pyonephrosis was suspected. While a ureteropelvic junction (UPJ) obstruction could not be ruled out, resolution with antibiotics makes it less likely. One dog had evidence of ureteral dilatation without evidence of ureteral calculi, likely secondary to ipsilateral nonobstructive nephrolith and secondary ureteritis.

Over the last 20 years, the Minnesota Urolith Center noted a decrease in the prevalence of struvite uroliths in dogs from 45.5% to 41.9%, with a corresponding increase in calcium oxalate uroliths from 40.9% to 45.2%.^3^, ^5^, ^40^, ^41^, ^42^ Recent evaluations of urolith submissions over the last decade found no change in the proportion of struvite‐containing submissions, possibly because of the effectiveness of dissolution diets and medical management.^37^, ^43^, ^44^ While most reported struvite uroliths exist in the lower urinary tract, they can also occur in the kidney and ureter of dogs. Upper urinary tract uroliths are less commonly reported and the majority of submitted struvite uroliths (>95%) are from the lower urinary tract.^45^ However, this may not accurately represent the prevalence, as many struvite uroliths (both upper and lower tract) are not removed and analyzed. Additionally, upper urinary tract calcium oxalate uroliths are often not removed, especially if they are nonobstructive. Approximately 29% to 33% of canine renal calculi and 42% of canine ureteral calculi are exclusively composed of struvite,^3^, ^5^, ^15^, ^21^, ^40^ with signalment, urinalysis, and urine culture results playing an important role in diagnosis.^2^, ^5^, ^46^, ^47^, ^48^

Treating nephroureterolithiasis in dogs now favors noninvasive approaches to reduce renal compromise, anesthesia time, recovery periods, and complications. Extracorporeal shockwave lithotripsy (ESWL),^17^, ^18^, ^49^ ENL,^19^ and burst wave lithotripsy^50^ are recognized as minimally invasive and effective for nondissolvable upper urinary tract uroliths.^2^ When possible, surgical removal should be avoided if uroliths can be dissolved, as we have documented with suspected struvite uroliths.

Results of our study indicate that long‐term dissolution treatment with a combination of an extended duration of diet and antibiotics successfully dissolved suspected struvite ureteroliths and nephroliths in all dogs. One dog had small, inconsequential, residual fragments that were consistent with calcium apatite, which failed to dissolve. The accurate prediction of struvite composition is supported by the high dissolution success rate, especially considering the large urolith size, minimizing the chance of undetected passage. Ureteral decompression was achieved with endoscopic double‐pigtail ureteral stent placement in all dogs with concurrent ureteral obstruction. The start of dissolution occurred at a median of 1.1 months, with complete ureteral urolith dissolution at a median 3.9 months (range, 1.5‐7.6), nephroliths at 5.3 months (range, 1.5‐7.6), and cystoliths at 0.87 months (range, 0.42‐5.9). One dog had residual uroliths of calcium apatite, not suitable for medical dissolution. Another dog developed recurrent nonobstructive nephroliths suspected to be struvite 12.3 months after dissolution, prompting reinstitution of dissolution treatment upon detection.

Most dogs showed clinical improvement, but many failed to present for the monthly follow‐up. One dog developed a highly resistant UTI with a different *Staphylococcus* spp. after discontinuing chronic antibiotic treatment after dissolution, without a stent. Sensitivity differed from the initial organism. Another dog had a prior resistant infection necessitating meropenem treatment for over 3 years, regardless of ureteral stent presence, before becoming infection‐free for 3 years, 5.5 years after urolith dissolution, with aggressive supportive measures. Humans are currently screened for asymptomatic bacteriuria before urological procedures and implants. If urine culture shows clinically relevant bacterial growth, or a colonized urolith is suspected, treatment based on susceptibility is recommended.^51^ Given the association of struvite urolith with urease‐producing bacteria, serial urine cultures during and after dissolution period was recommended to monitor for recurrence and possible need for restarting medical management. Recrudescence of bacteria during the dissolution period is common because of the bacteria within the nidus of struvite uroliths. Monitoring helps ensure no resistant pathogen is present that could hinder dissolution or necessitate a change in antibiotic protocol.

The median hospitalization time was 2 days. All hospitalized (n = 5) and outpatient (n = 1) dogs received broad‐spectrum antibiotics upon discharge, adjusted based on culture and sensitivity data. Enrofloxacin was used solely in 3/6 cases, amoxicillin/clavulanic acid in 1/6, and a combination of both in 2/6 cases. One dog needed meropenem (SC), then nitrofurantoin and enrofloxacin because of multidrug‐resistant pathogens. Financial constraints led 1 dog to transition from combination treatment to cefpodoxime. Another shifted from enrofloxacin to cefpodoxime because of persistent UTI despite appropriate initial antibiotic selection. Transition to cefpodoxime in both dogs was guided by urine culture and sensitivity and did not influence resistance patterns on subsequent cultures. Three dogs developed additional UTIs during dissolution, attributed to poor compliance and premature treatment cessation despite improved signs. Identified pathogens included *S schleferi*, *E coli*, *or Enterococcus*, treated with tailored antibiotics based on culture data. One dog underwent 2 ureteral stent exchanges because of recurrent infections and new urolith formation.

Limitations include the retrospective design and inconsistent follow‐up, hindering accurate dissolution timing determination. Treatment involved strict dissolution diet, concurrent antibiotics, and ureteral stent decompression when needed. Long‐term management varied among referring veterinarians and was not standardized after initial clinician‐led care; all dogs were prescribed dissolution diet (Urinary SO, Royal Canin Veterinary Diets, St. Charles, Missouri; C/D Multicare, Hill's Prescription Diets, Topeka, Kansas) One used a homemade nutritionist formulation because of commercial diet intolerance. Acidification medications were not used. Urolith dissolution was successful in all cases.

Considering a dog's signalment, urine sediment, culture, urolith radiopacity, and shape can successfully predict urolith composition, aiding in decision to pursue medical dissolution, which offers a less invasive approach. Medical management can effectively treat upper urinary tract uroliths, even with substantial urolith burdens and aggressive surgical urolith removal should be discouraged.

## CONFLICT OF INTEREST DECLARATION

Authors declare no conflict of interest.

## OFF‐LABEL ANTIMICROBIAL DECLARATION

Authors declare no off‐label use of antimicrobials.

## INSTITUTIONAL ANIMAL CARE AND USE COMMITTEE (IACUC) OR OTHER APPROVAL DECLARATION

Authors declare no IACUC or other approval was needed.

## HUMAN ETHICS APPROVAL DECLARATION

Authors declare human ethics approval was not needed for this study.

## Supporting information


**Data S1:** Supporting Information.
