# Supplementary material for: Medical dissolution of presumptive upper urinary tract struvite uroliths in 6 dogs (2012‐2018)
Source: J Vet Intern Med. 2024 Oct 5;38(6):3095–104. doi: 10.1111/jvim.17204 (PMC11586565; doi:10.1111/jvim.17204)
Supplement: Supplementary file 1 — Data S1: Supporting Information. [file JVIM-38-3095-s001.docx]

**Procedures:**

Pre-operative use and duration of IV fluid and antimicrobial therapy were not standardized due to many dogs receiving medical management prior to referral. The typical anesthesia protocol included premedication with oxymorphone (0.1 mg/kg [0.045 mg/lb], IM) and induction of general anesthesia with propofol (3 mg/kg [1.36 mg/lb], IV) or a combination of etomidate (1 to 2 mg/kg [0.45 to 0.9 mg/lb], IV) and midazolam (0.25 to 0.5 mg/kg [0.11 to 0.23 mg/lb], IV), followed by endotracheal intubation and maintenance of anesthesia with isoflurane in oxygen and a fentanyl constant rate infusion (0.35 to 0.7 μg/kg/min [0.16 to 0.32 μg/lb/min], IV). If hypotension occurred during anesthesia, dopamine (5 to 12 μg/kg/min (2.3 to 5.5 μg/lb/min) was administered as a constant rate infusion. Once anesthetized, dogs were positioned in dorsal recumbency. The abdomen and vulva were clipped of hair and aseptically prepared, and the vulva was lavaged with dilute chlorohexidine solution. The entire abdomen and vulva were draped in standard fashion. Dogs were administered cefazolin antimicrobial (22mg/kg [10 mg/lb], IV] every 2 hours throughout the procedure if they were not currently receiving antimicrobials with gram-positive organism coverage.
